# Dynamics of the *Phanerochaete carnosa* transcriptome during growth on aspen and spruce

**DOI:** 10.1186/s12864-018-5210-z

**Published:** 2018-11-13

**Authors:** E. Jurak, H. Suzuki, G. van Erven, J. A. Gandier, P. Wong, K. Chan, C. Y. Ho, Y. Gong, E. Tillier, M.-N. Rosso, M. A. Kabel, S. Miyauchi, E. R. Master

**Affiliations:** 1 Department of Bioproducts and Biosystems, Aalto University, Espoo, Finland; 2 Department of Aquatic Biotechnology and Bioproduct Engineering, Groningen, The Netherlands; 3 Department of Chemical Engineering and Applied Chemistry, University of Toronto, Toronto, Canada; 4 Wageningen University, Laboratory of Food Chemistry, Bornse Weilanden 9, 6708 WG Wageningen, The Netherlands; 5 Department of Medical Biophysics, University of Toronto, Toronto, Canada; 6 Samuel Lunenfeld Research Institute, Mount Sinai Hospital, Toronto, Canada; 7 Centre for the Analysis of Genome Evolution and Function, University of Toronto, Toronto, Canada; 8 Laboratory of Excellence ARBRE, INRA, Nancy, Lorraine France; 9 Aix-Marseille Université, INRA, UMR1163, Biodiversité et Biotechnologie Fongiques, Marseille, France

**Keywords:** *Phanerochaete carnosa*, Transcriptomics, Carbohydrate active enzymes, Lignocellulose conversions, Loosenins, Hydrophobins

## Abstract

**Background:**

The basidiomycete *Phanerochaete carnosa* is a white-rot species that has been mainly isolated from coniferous softwood. Given the particular recalcitrance of softwoods to bioconversion, we conducted a comparative transcriptomic analysis of *P. carnosa* following growth on wood powder from one softwood (spruce; *Picea glauca)* and one hardwood (aspen; *Populus tremuloides*). *P. carnosa* was grown on each substrate for over one month, and mycelia were harvested at five time points for total RNA sequencing. Residual wood powder was also analyzed for total sugar and lignin composition.

**Results:**

Following a slightly longer lag phase of growth on spruce, radial expansion of the *P. carnosa* colony was similar on spruce and aspen. Consistent with this observation, the pattern of gene expression by *P. carnosa* on each substrate converged following the initial adaptation. On both substrates, highest transcript abundances were attributed to genes predicted to encode manganese peroxidases (MnP), along with auxiliary activities from carbohydrate-active enzyme (CAZy) families AA3 and AA5. In addition, a lytic polysaccharide monooxygenase from family AA9 was steadily expressed throughout growth on both substrates. P450 sequences from clans CPY52 and CYP64 accounted for 50% or more of the most highly expressed P450s, which were also the P450 clans that were expanded in the *P. carnosa* genome relative to other white-rot fungi.

**Conclusions:**

The inclusion of five growth points and two wood substrates was important to revealing differences in the expression profiles of specific sequences within large glycoside hydrolase families (e.g., GH5 and GH16), and permitted co-expression analyses that identified new targets for study, including non-catalytic proteins and proteins with unknown function.

**Electronic supplementary material:**

The online version of this article (10.1186/s12864-018-5210-z) contains supplementary material, which is available to authorized users.

## Background

Fungi from the phylum Basidiomycota, class Agaricomycetes, include ectomycorrhizal fungi, saprotrophs, as well as efficient wood (lignocellulose) degraders. White-rot fungi of the orders Agaricales and Polyporales are especially adept wood-degraders. Accordingly, these fungi have been the focus of studies aimed at the bioconversion of major lignocellulose components, including strategies to hydrolyze cellulose and hemicelluloses to monosaccharides for fermentation to fuels and chemicals. Since the first publication of the *Phanerochaete chrysosporium* genome in 2004 [1], the number of Basidiomycota genome sequences has increased to several hundred (https://jgi.doe.gov/) [2]. Among these, *Phanerochaete carnosa* represents a white-rot that grows on both deciduous (hardwood) and coniferous (softwood) fibre, but has been almost exclusively isolated from softwoods [3]. Its genome was sequenced in 2012 [4], confirming *P. carnosa* encodes a full complement of carbohydrate-active enzymes (CAZymes) for lignocellulose conversion and revealing a large contingent of predicted cytochrome P450 monooxygenases.

Coniferous trees are the predominant form of renewable biomass in the northern hemisphere; however, it is especially recalcitrant to bioprocess technologies. The recalcitrance of coniferous wood has been attributed to the higher lignin content, smaller pore size, and fewer hemicellulose-derived acetyl groups in comparison to deciduous woods [5, 6]. Challenges linked to softwood bioconversion have motivated studies that investigate gene and protein expression by white-rot fungi that grow on coniferous wood [7–15]. In general, corresponding studies show particularly high expression of lignolytic enzymes (e.g., lignin peroxidases (LiPs) and manganese peroxidases (MnPs)) and lytic polysaccharide monooxygenases (LPMOs); comparatively high expression of glycoside hydrolases (GHs) from families GH5, GH6, GH7, GH10, GH12, GH28, GH43, and GH131 have also been repeatedly reported. So far, such comparative analyses mainly consider either multiple substrates or multiple time points on a single wood species. Accordingly, time and substrate dependent influences on the expression of lignocellulose degrading activities remain unclear.

Herein, we apply a transcriptomic approach to track gene expression by *P. carnosa* over five growth points on heartwood of white spruce (*Picea glauca*) and trembling aspen (*Populus tremuloides*). Earlier transcriptomic analyses of *P. carnosa* grown on fir, pine, spruce and maple wood preparations show high transcript abundances corresponding to specific MnPs and LPMOs [15]; however, impacts of biomass conversion on resulting gene expression profiles could not be gleaned from the single time point included in that study. By evaluating the impact of both wood substrate and time on the *P. carnosa* transcriptome, we can identify specific enzymes, enzyme sub-families, and novel activities best correlated to plant biomass degradation and most critical to early versus late stages of wood decay.

## Results

### Growth on wood substrates

Mycelia samples were harvested at five equivalent radial distances (between 2 and 9 cm) from the center of solid-state cultivations on aspen and spruce. In this way, we could evaluate changes in the gene expression profiles of *P. carnosa* over a comparable extent of radial growth on the two wood substrates, and ensure in both cases that sufficient quantities of mycelia would be collected for RNA extraction. The resulting growth points (GP) 1–5 corresponded to 7 to 23 days of cultivation on aspen, and 13 to 30 days of cultivation on spruce. While growth was initially slower on spruce, the radial growth rate of *P. carnosa* was independent of substrate following GP1 (Additional file 1). This suggests that a longer adaptation period was required to establish growth on spruce; however, following the adaptation period, *P. carnosa* grew similarly on both spruce and aspen.

Hierarchical clustering of transcriptome profiles were consistent with the growth patterns, where following the initial lag phase on spruce, similar transcriptome patterns were obtained from cultivations on spruce and aspen (Additional file 2). Notably, the relative carbohydrate composition was similar throughout growth of *P. carnosa* on both wood substrates, consistent with non-selective consumption of corresponding monosaccharides (Additional file 3). On the other hand, slight but significant loss of lignin was measured only from aspen (Additional files 4 and 5). Herein, wood samples were ball milled prior to fungal cultivation, which was expected to increase the accessibility of the wood substrates and permit comparative transcriptome analyses that reveal fungal responses to differences in wood fibre composition uncoupled from differences in wood fibre structure.

### Transcriptome profiles of sequences predicted to encode lignocellulose-active CAZymes

**Fig. 1:**
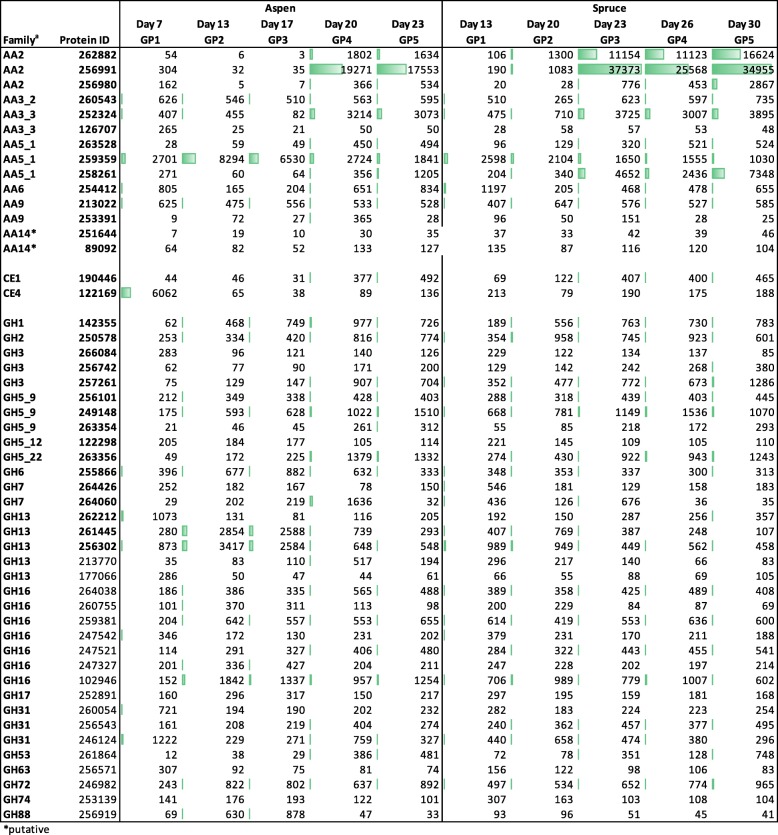
CAZymes having > 2.5 times the transcript abundance of the median CPM per growth point. Abundances (CPM) are specified and represented by the relative length of the data bars. ^a^ Assignments based on the carbohydrate-active enzyme database (http://www.cazy.org), predicted to encode lignocellulose-active enzymes. *putative CAZy family assignment

Considering all 13,937 genes encoded by the *P. carnosa* genome [4], sequences having highest transcript abundance on both wood substrates were mainly household metabolism regulating genes, transporters, MnPs (Phaca262882, Phaca256991) and uncharacterized sequences (Additional file 6). The 246 sequences encoding carbohydrate active enzymes (http://www.cazy.org; CAZymes; Additional file 7) were considered in more detail, given they encode proteins predicted to contribute to lignocellulose conversion. This analysis uncovered a core set of CAZyme sequences present at high transcript abundance for both cultivation conditions (Fig. 1), consistent with similar extents of growth observed on both wood substrates following the initial lag phase on spruce.

Of the seven MnPs encoded by *P. carnosa*, transcript sequences corresponding to two MnPs (Phaca262882, Phaca256991) were 5 to 10 times more abundant than any other predicted CAZyme (Fig. 1). These same sequences were among the 30 most abundant transcripts expressed by *P. carnosa* during growth on maple, fir, pine, and spruce [15], confirming the biological relevance of these particular MnPs for conversion of lignin present in both deciduous and coniferous wood. In addition to MnPs, transcripts predicted to encode enzymes that provide H_2_O_2_ (required for MnP activity), including glyoxal oxidases (GLOX), GLOX/related copper radical oxidases (CRO) and alcohol (AOX) oxidases [16, 17] were also among the top 25 highly expressed sequences. Of these, transcript sequences encoding two AA3 alcohol oxidases (Phaca260543 and Phaca252324) and one AA5_1 oxidase (GLOX; Phaca259359) followed the MnP’s in terms of relative abundance of CAZyme sequences. Also of note, transcripts encoding two distantly related AA5_1 oxidases displayed divergent substrate-dependent expression patterns. Specifically, Phaca259359 transcript abundance was higher on aspen than spruce, whereas the reverse pattern was observed for Phaca258261. Phaca258261 is phylogenetically related to glyoxal oxidases implicated in H_2_O_2_ production [16]. By contrast, Phaca259359 shares 84% sequence identity to CRO2 encoded by *P. chrysoporium*, which displays a distinct substrate preference relative to glyoxal oxidases [17], and whose biological function remains unclear.

Of the 11 family AA9 LPMOs encoded by *P. carnosa*, transcript levels corresponding to Phaca213022 were 5 to 10 times higher than the second most highly expressed AA9 sequence (Phaca253391) (Fig. 1). Moreover, Phaca213022 transcript abundance was comparatively steady over time in both aspen and spruce cultivations. The discovery of the family AA14 LPMO from the basidiomycete *Trametes coccinea* (i.e., PcAA14A) [18], prompted us to search for possible AA14 members in the *P. carnosa* genome. PcAA14A catalyzes the oxidative cleavage of xylan-coated cellulose; two potential AA14 members were identified herein, namely Phaca251644 (70.7% identity to PcAA14A) and Phaca89092 (56.8% identity to PcAA14A). Although levels were low, in both cases transcript abundances increased between the first and last growth point on aspen; by contrast, transcript abundances were steady on spruce (Fig. 1).

Abundances of transcripts predicted to encode glycoside hydrolases, carbohydrate esterases, and polysaccharide lyases were generally lower than those predicted to encode auxiliary activities. Of the 24 family GH5 sequences and 24 GH16 sequences encoded by *P. carnosa*, transcript abundances for 5 GH5s and 7 GH16s were at least 2.5 times above the median CPM value for at least one growth point (Fig. 1). Among the GH5s, three belonged to subfamily GH5_9 and one belonged to subfamily GH5_22, which are predicted to act on fungal and plant polysaccharides, respectively [19]. The transcript abundance of four GH16 sequences also increased over time, particularly during growth of *P. carnosa* on aspen (Phaca264038 Phaca259381, Phaca247521, Phaca102946). However, functional prediction for GH16 members remains complicated by the diverse activities and biological roles attributed to this CAZy family [20].

Levels of transcripts encoding the five predicted GH10 xylanases and three GH12 endoglucanases encoded by *P. carnosa*, as well as polysaccharide lyases and GH28 enzymes contributing to pectin degradation, were comparatively low and steady on both aspen and spruce (Additional file 8). By contrast, transcript abundances increased over the cultivation for sequences in families CE1, GH2, and GH3, which are known to include enzymes that target plant cell wall carbohydrates (Fig. 1; Additional file 7). Increase in transcript abundance was not observed, however, for the sole predicted GH6 cellobiohydrolase and the two most highly expressed GH7 cellobiohydrolases encoded by *P. carnosa*. Instead, corresponding transcript abundances were dependent on both time and substrate (Fig. 1).

### Transcriptome profiles of sequences predicted to encode P450 monooxygenases

Cytochrome P450 monooxygenases have been implicated in the degradation of small lignin fragments and other aromatic compounds, and could thus facilitate fungal growth on wood by detoxifying lignin degradation products as well as aromatic extractives [21]. The *P. carnosa* genome comprises 266 genes predicted to encode cytochrome P450 monooxygenases, nearly twice the number encoded by *P. chrysosporium* [4].

**Fig. 2:**
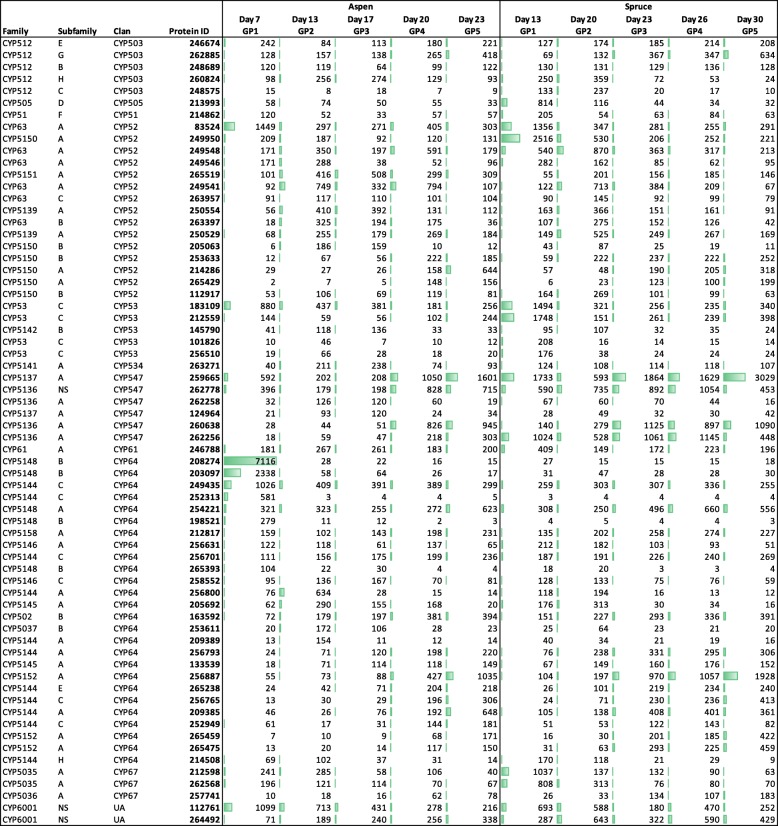
P450s having > 2.5 times the transcript abundance of the median CPM per growth point. Abundances (CPM) are specified and represented by the relative length of the data bars

Patterns of P450 transcript abundance were generally similar during growth of *P. carnosa* on the two wood substrates, where 50% or more of the most highly expressed P450 mainly grouped in clans CYP52 and CYP64 (Fig. 2). P450s belonging to clan CYP64 were also highly expressed in *P. coccineus* following cultivation on pine and aspen [9]. Of note, clans CYP52 and CYP64 accounted for most of the P450 sequence expansion in *P. carnosa* compared to *P. chrysosporium*. Transcript abundances were highest, however, for two sequences corresponding to clan CYP547 (Phaca260638 and Phaca259665).

### Co-expression analyses

**Fig. 3:**
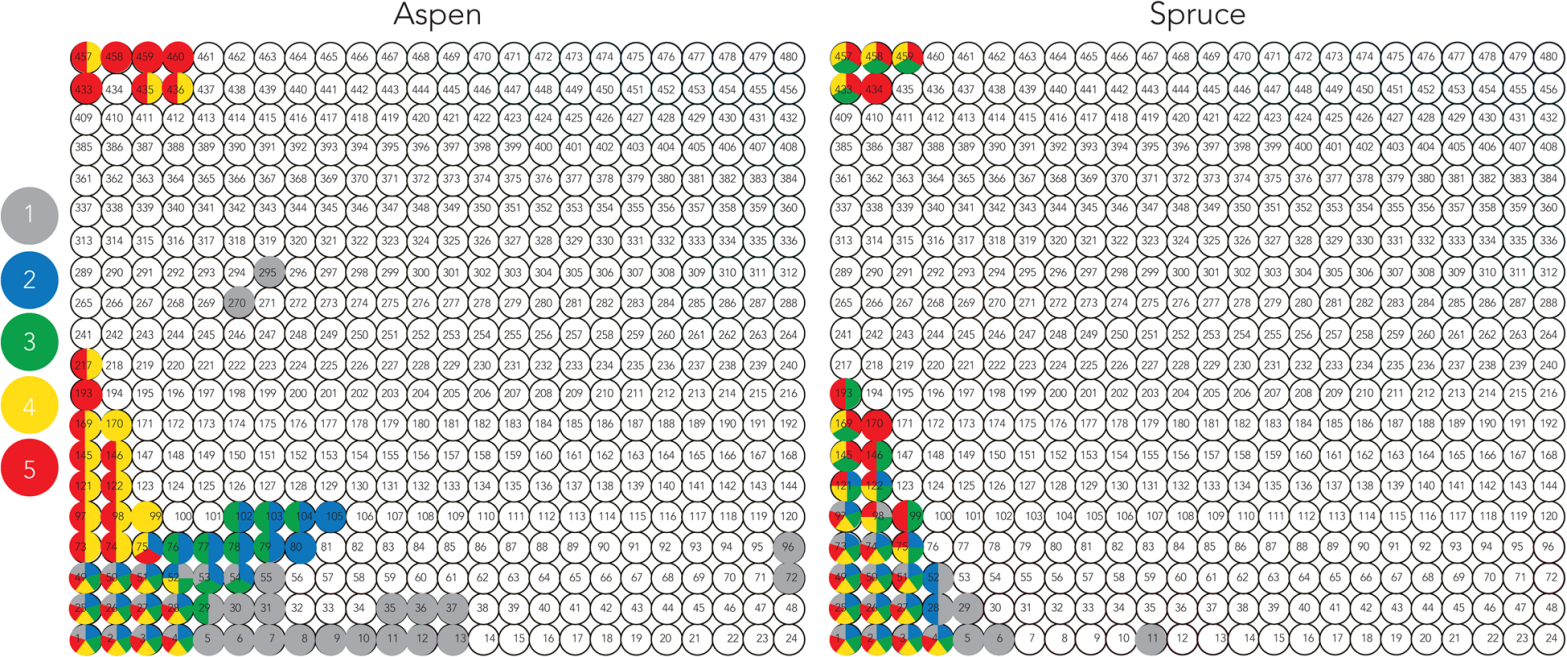
Tatami maps showing clusters of high/differential transcriptions following growth on aspen and spruce. Nodes are coloured based on high/differential transcription at the growth point 1 to 5. The condition-specific nodes were determined according to two criteria: 1) > 10.2 mean log2 reads that corresponds to above 95th percentile of the transcription level of the all genes used for the transcriptomic model; and 2) > 2 log2 fold differences of each growth point against growth point 1. Node identification is labelled (1 to 480). Co-transcribed CAZymes encoded by *P. carnosa* that correspond to specific nodes are listed in Additional file 8

The consistency of the transcriptomic data permitted the construction of transcriptomic models using the SHIN+GO pipeline (Additional files 2, 9, 10) [22]. Resulting self-organizing maps (SOM) group genes with similar transcriptional patterns and form nodes arranged as so-called Tatami maps (Additional files 11 and 12; node number and composition listed in Additional file 8). Within a Tatami map, nodes in close proximity contain genes with relatively similar transcriptional patterns (Fig. 3).

The co-expression analyses identified groups of gene products that may operate together. For example, MnPs require a source of H_2_O_2_, which can be generated by family AA3 and AA5 oxidases. Clustering of the MnP (Phaca256991) and a AA3_3 oxidase (Phaca252324) within node 49, and the neighbouring positions of nodes 145, 169 and 170 (Fig. 3) that comprise the most highly expressed MnP (Phaca262882; node 145), along with a family AA3_3 oxidase (Phaca121157; node 170), and a family AA5_1 oxidase (Phaca263528; node 169), predict these specific auxiliary enzymes may act in concert to transform lignin.

Considering the profile of transcripts encoding P450 monooxygenases, co-expression analysis underscored the transition over time from sequences belonging to many P450 clans to sequences predominately from clan CYP52 and clan CYP64, which are also expanded in the *P. carnosa* genome (nodes 145, 193, and 457, Fig. 3). Moreover, nearly half of nodes including a P450 sequence also included predicted glutathione-S-transferases, which are also believed to play a role in the detoxification of compounds released during fungal growth on lignocellulosic materials [23, 24] (Additional file 8).

**Fig. 4:**
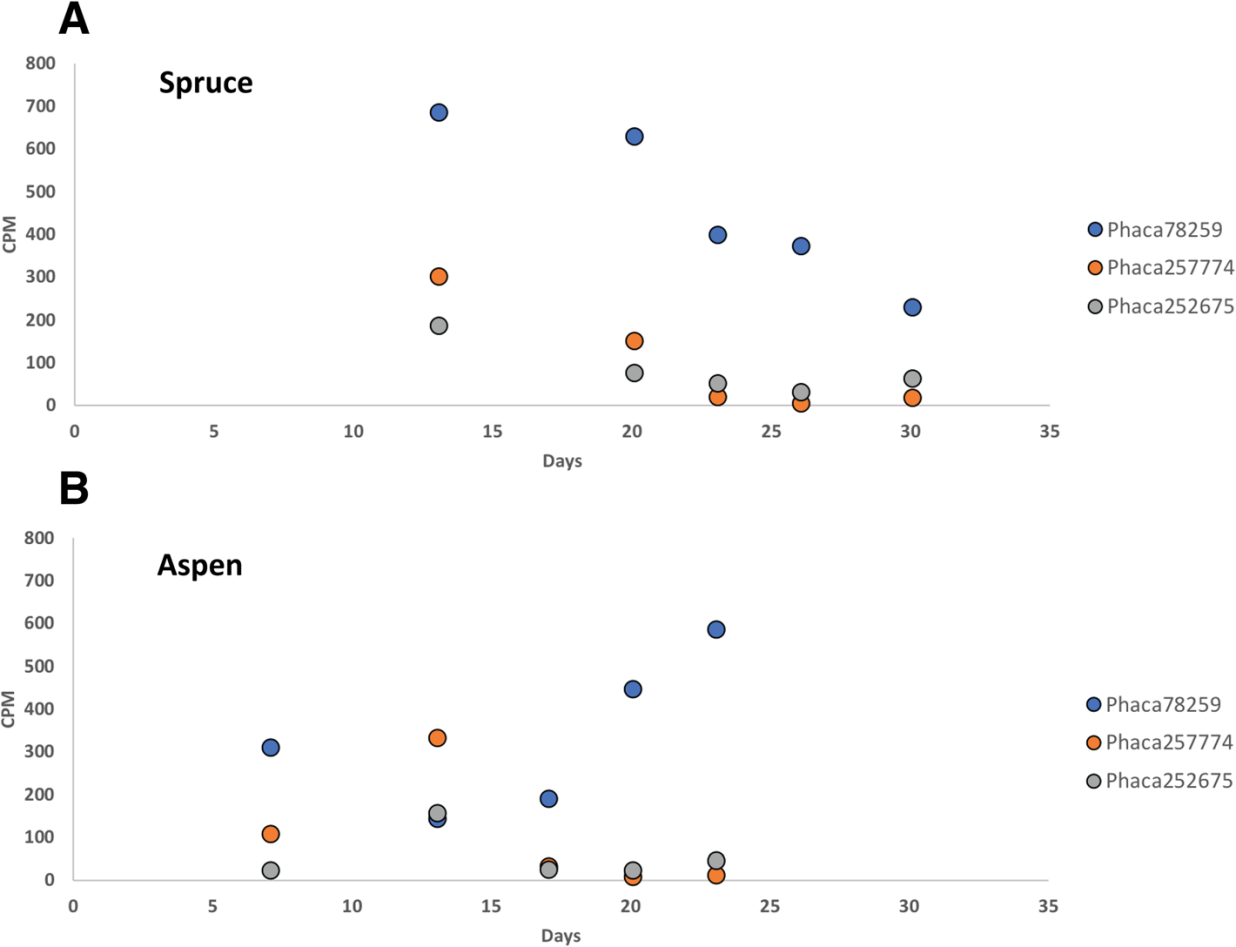
Transcript abundance over time for highly expressed hydrophobins on (**a**) aspen and (**b**) spruce. CPM values are given for each growth point on both substrates

Co-expression analyses was also used herein to identify non-catalytic proteins, namely loosenins and hydrophobins, that co-express with known CAZymes and may influence fungal growth on lignocellulosic substrates. Briefly, loosenins are single domain proteins that adopt a DPBB fold homologous to domain 1 of expansins [25, 26]. On the other hand, hydrophobins are surface active proteins secreted by filamentous fungi, which are subdivided into two classes, I and II [27, 28]. Whereas some loosenins show cellulose disruption activity [25], hydrophobin films can reverse the wettability of solid surfaces; it has also been suggested that such films could play roles in recruiting enzymes to substrates [29]. The *P. carnosa* genome is predicted to encode for twelve loosenin-like proteins (LOOL), along with one sequence that is distantly related to plant expansins (DREX) [30]. Transcripts of all thirteen of these genes were detected. Of these, transcripts encoding LOOL2 (Phaca255931) were most abundant; increasing to levels comparable to AA3 oxidases and various glycoside hydrolases at day 13 on both spruce and aspen (CPM values of 236 and 469, respectively; Additional file 6). Co-expression analyses clustered LOOL2 with a predicted family CE9 N-acetyl-glucosamine 6-phosphate deacetylase, suggesting a role in fungal cell wall morphogenesis (Additional file 8). All 13 hydrophobin sequences predicted from the *P. carnosa* genome encode Class I proteins and were detected at the transcript level. Of these, transcript abundances for three sequences were at least 100 CPM for one or more growth points (Fig. 4; Additional file 6). In particular, the transcript abundance of Phaca78259 was up to 12 and 38 times higher than Phaca25774 and Phaca252675, reaching 590 CPM on aspen and 690 CPM on spruce. The transcript profile of Phaca78259 was also reversed on aspen versus spruce, where abundances generally increased and decreased over time, respectively (Fig. 4). Notably, the Phaca78259 transcript profile clustered into node 26 (Fig. 3), which also includes the most highly expressed LPMO (Phaca213022) along with two GH families that likely contribute to fungal cell wall modification, namely a putative β-1,3-glucanosyltransglycosylase from family GH72 and β-1,3-glucanase from family GH128 [31] (Additional file 8).

**Fig. 5:**
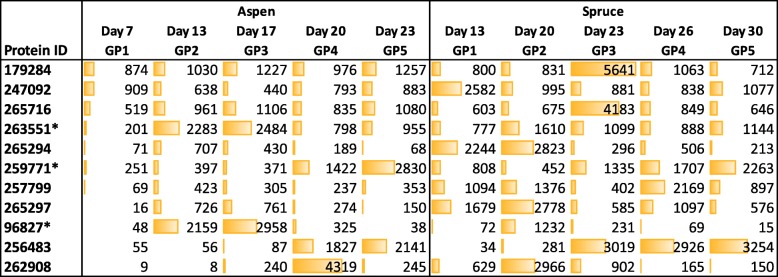
Most abundant transcripts encoding proteins with unknown function that cluster with known CAZymes. Abundances (CPM) are specified and represented by the relative length of the data bars. *predicted signal peptide

Lastly, the co-expression analyses performed herein were used to identify sequences with unknown function that co-expressed with differentially and highly expressed CAZymes. Eleven highly and differentially expressed sequences with unknown function that co-expressed with annotated CAZyme sequences were identified (Fig. 5). Of these, three were predicted to encode a signal for secretion; moreover, Phaca259771 is predicted to encode a cupredoxin domain with the ability to bind copper. Transcript abundances for both Phaca259771 and Phaca256483 increased over time, and clustered into node 49 and 145, respectively, which also contain the most highly expressed MnPs (i.e., Phaca256991 and Phaca262882, respectively). Together, the presence of the predicted signal sequence for secretion, cupredoxin domain, and co-expression with a highly expressed MnP (Phaca256991) suggests that the protein with unknown function, Phaca259771, may in fact contribute to MnP action through, for example, H_2_O_2_ production.

## Discussion

The wood samples used to cultivate *P. carnosa* were ball milled to increase the accessibility of the wood substrates and permit comparative transcriptome analyses that reveal fungal responses to differences in wood fibre composition uncoupled from differences in wood fibre structure. While this approach was expected to reduce the requirement for low molecular weight molecules thought to promote incipient stages of fungal growth on intact wood samples [32–34], the overall transcriptome patterns generated by *P. carnosa* during growth on spruce and aspen were similar despite differences in hemicellulose, lignin, and extractive contents in these wood substrates. Recent studies of other fungi report analogous findings. For example, *Fomitopsis pinicola* elicits similar patterns of CAZyme gene expression following growth on aspen, pine, and spruce [35]. Instead, more significant differences were correlated to wood sample preparation (e.g., wood wafers versus wood powder) [35], underpinning the importance of 1) uncoupling fibre structure from composition when the aim is to compare impacts of compositional differences, and 2) considering the mode of fibre pretreatment when the aim is to improve enzyme formulations for biomass processing.

Transcriptomic analysis of *P. carnosa* at five growth points on two substrates uncovered expression patterns for transcripts present at low abundance, which can be used to guide sequence selections for functional characterization. For example, transcripts encoding family GH16 sequences were grouped into those that were most abundant at initial or late stages of fungal growth, or else steadily expressed over the cultivation period (Fig. 3; Additional file 8). Functional predictions of GH16 sequences is complicated by the several activities that have been attributed to this CAZy family, including xyloglucan transglycosylase activity observed for plant GH16s, and lichenase, laminarinase, and agarase activities observed for microbial GH16s [20]. Diverse biological processes have also been attributed to these enzymes, including fungal cell wall synthesis [36, 37], and hydrolysis of β-glucans in endosperm cell walls of barley and other cereals [38]. Of particular note, the most abundant GH16 transcript encoded by *P. carnosa* during cultivation on wood (Phaca102946) shares less than 30% sequence identity with currently characterized fungal GH16s.

The expanded set of P450 genes in *P. carnosa* could enable its growth on heartwood and coniferous wood in general, which is typically characterized by comparatively high lignin and extractive content [4, 8]. Remarkably, over 50% of the most highly expressed P450 mainly grouped in clans CYP52 and CYP64, which also account for most of the P450 sequence expansion in *P. carnosa*. The current transcriptome analyses thus strengthen the hypothesis that both clans CYP64 and CYP52 play an important role in enabling *P. carnosa* to colonize and grow on heartwood tissue of both deciduous and coniferous sources, while at the same time, reveal the likely relevance of clan CYP547.

Co-expression analyses can identify groups of gene products that may operate together. Herein, a differentially expressed hydrophobin sequence was shown to group with known CAZymes, including sequences belonging to families AA9 and GH128. Certainly, the role that hydrophobins may play in interacting with and accessing lignocellulosic substrates remains unclear. Still, other studies are beginning to note the expression of these proteins during fungal cultivation on wood. For example, Couturier et al., [9] report 500 times higher expression of a predicted hydrophobin during *P. coccineus* cultivation on pine and aspen compared to maltose. Likewise, Kuuskeri et al., [10] found hydrophobin transcripts amongst those most upregulated in *P. radiata* during growth on wood. Co-expression analyses also identified a protein with unknown function containing a predicted cupredoxin domain (Phaca259771), which grouped with highly expressed MnP sequences. A cupredoxin containing protein of unknown function was also identified through transcriptomic analysis of *P. chrysosporium* grown on spruce [8]; however, the sequence identity to Phaca259771 is only 34%. Notably, comparisons between highly-expressed proteins with unknown function identified herein, and those highlighted in earlier transcriptome analyses of softwood-degrading basidiomycetes [8, 9, 22, 39], did not reveal a core set of related sequences.

## Conclusions

Following an initial lag phase during growth on spruce compared to aspen, the transcriptome elicited by *P. carnosa* were similar on both wood substrates. For both cultivation conditions, the most abundant transcript encoded the same MnP (Phaca256991), followed by AA3 and AA5_1 oxidases that may generate the H_2_O_2_ required for MnP activity. Approximately 25% of the identified P450 monooxygenases encoded by *P. carnosa* were also marked as highly expressed during growth on aspen and spruce. These mainly belonged to clans CYP52 and CYP64, which are also expanded in the *P. carnosa* genome.

Overall, transcript abundances for glycoside hydrolases and carbohydrate esterases were lower than those encoding auxiliary oxidoreducases. Of these, transcripts encoding GH2, GH5, GH6, GH7, GH16, and CE1, were among the most highly expressed sequences predicted to encode plant biomass degrading enzymes. Similar expression profiles were observed for other softwood-degrading white-rot fungi, including *Dichomitus squalens*, *Phlebia radiata*, and *Obba rivulosa*, and *Pycnoporus coccineus* [9–13]. The current study further showed that despite known differences in the compositions of spruce and aspen, *P. carnosa* produces a similar profile of CAZymes transcripts when grown on these substrates. This observation is consistent with recent studies that underscore the contribution of wood sample preparation (e.g., wood wafers versus wood powder) [35], in addition to specific tree species, age, and wood tissue on the expression of CAZymes by wood-degrading fungi.

All differentially expressed transcripts encoding carbohydrate-active enzymes belonged to the core set of plant biomass degrading enzymes previously predicted through comparative analysis of basidiomycete transcriptomes [40]. The resolution afforded by the multiple growth points included herein, however, revealed distinct expression profiles of GH families having relatively low transcript abundance yet recognized roles in plant polysaccharide conversion. These results can be used to guide the selection of *P. carnosa* sequences for functional characterization, which is especially important when considering comparatively large CAZyme families (e.g., GH5 and GH16).

The inclusion of several growth points in the current study also permitted detailed cluster analysis of co-expressed transcripts, which uncovered enzymes that may operate in concert, including MnPs and carbohydrate oxidases from families AA2 and AA3, as well as predicted P450 monooxygenases and glutathione S-transferases. Furthermore, co-expression analyses uncovered non-catalytic proteins and proteins with unknown function that could contribute to lignocellulose conversion; in particular Phaca259771, which is predicted to encode a cupredoxin domain. However, the low sequence identity of highly expressed transcripts encoding unclassified proteins from diverse lignocellulose-degrading fungi, further underscores the importance of comparable cultivation methods to expanding the core set of carbohydrate-active enzymes for lignocellulose conversion.

## Methods

### Fungal cultivation

*Phanerochaete carnosa* strain HHB-10118-sp was obtained from the U.S. Department of Agriculture (USDA) Forest Products Laboratory (Madison, WI) and maintained on YMPG agar plates (2 g yeast extract, 10 g malt extract, 2 g peptone, 10 g glucose, 2 g KH_2_PO_4_, 1 g MgSO_4_·7H_2_O, and 15 g agar per 1 L in H_2_O) as previously described [4]. Wood samples were obtained from New Brunswick, Canada, where a 50 cm bolt at 80 cm and 130 cm trunk heights were cut from trembling aspen (*Populus tremuloides*) and white spruce (*Picea glauca*); heartwood sections were then separated, air-dried, and then ground in a Wiley mill (Thomas scientific, NJ, USA) followed by planetary ball mill [4, 30]. Approximately 4 g of wood particles were distributed as a thin layer in Petri dishes, autoclaved, and then supplemented with 20 mL of B3 buffer [14, 30]. To maximize the reproducibility of fungal growth patterns, a 1 cm diameter agar plug taken from the growing edge of *P. carnosa* cultivated on YMPG agar plates was directly transferred to the center of each plate containing wood particles, and incubated at 27 °C under stationary conditions. As performed previously [14, 15, 30], all mycelia for 2 cm colonies, and the central 4 cm of colonies reaching 5, 6, 8, and 9 cm in diameter, were harvested and then stored at − 80 °C prior to RNA extraction and wood analyses. By sampling mycelia from the centre of the growing colony rather than the growing edge of the colony, corresponding transcriptomes were more likely to reflect responses to potentially changing substrate composition resulting from fungal growth. Moreover, this approach to fungal cultivation yielded similar and sufficient quantities of RNA for sequencing, and at the same time, ensured reproducible harvesting of biological replicates. Three replicate cultivations were prepared for each colony size (Additional file 1).

### RNA extraction and sequencing

Total RNA was isolated from frozen mycelia using the Plant/Fungi Total RNA Purification Kit (Norgen Biotek) according to the manufacturer’s instructions. The quality and quantity of purified RNA were monitored using a Bioanalyzer (Agilent Technologies). A portion of purified total RNA was used for first strand cDNA synthesis using RevertAid reverse transcriptase (Thermo Scientific), and the reproducibility between three biological replicates was verified by quantitative reverse-transcription PCR (qRT-PCR) for a manganese peroxidase (MnP, Phaca262882) and chitin synthase gene (Chs, Phaca257626) [15]. Two of the three biological replicates for each cultivation were then randomly selected, and total RNA from those replicates were utilized for independent RNA sequencing.

The cDNA library was prepared using TruSeq RNA Sample Prep Kit v2 (Illumina). Briefly, 1 μg of high quality total RNA was used to generate the cDNA library having an average fragment size of 350–400 bp. The quality of the barcoded library was checked using a Bioanalyzer and quantified by qPCR using KAPA SYBR FAST Universal 2X qPCR Master Mix (Kapa Biosystems) running in 7900HT Fast Real Time PCR System (Applied Biosystems) [30]. The cDNA libraries were then loaded on a flowcell for cluster generation using c-Bot and TruSeq PE Cluster Kit v3 (Illumina). Sequencing was performed using a HiSeq2000 system and the TruSeq SBS Kit v3 (pair-ended 200 cycles, Illumina). 100 bp pair-ends were generated. The real-time base call (.bcl) files were converted to fastq files using CASAVA 1.8.2 (Illumina, on CentOS 6.0 data storage and computation linux servers at the Sequencing Facility of the Lunenfeld-Tanenbaum Research Institute, Mount Sinai Hospital, Toronto, Canada), and then aligned to JGI *P. carnosa* gene models (v1.0, http://genome.jgi-psf.org/Phaca1/Phaca1.home.html) with the Novoalign software (Novocraft) [15, 30]). Raw sequence data were deposited to the Sequence Read Archive (SRA accession: SRP151360).

### Bioinformatic analyses

#### Normalization of data and log2 fold differences of genes

Read counts of gene models were input into edgeR [41] for CPM (count per million) conversion and differential expression analysis; the consistency of expression values was verified by qRT-PCR and the reproducibility of CPM values between two biological replicates was verified using scatter plots [30]. For construction of transcriptomic models, the following procedures were performed. The log2 fold difference of the gene expression between time points was calculated with R package DESeq2 [42]. Genes with statistical significance were selected based on FDR (false discovery rate) adjusted *p* value < 0.05. Normalized read counts of the genes were also produced with DESeq2, which were subsequently log2 transformed. The consistency of normalized transcription from all biological replicates was confirmed by visualizing the distribution of read counts (Additional file 9). The expression of 27 housekeeping genes (NADH dehydrogenase and chitin synthase) under all conditions was investigated for the consistency of fungal growth (Additional file 10). A total of 11,796 genes having more than averaged five reads per condition were selected for constructing transcriptomic models.

#### Correlation among biological replicates

Spearman’s rank correlation was calculated with normalized read counts from the biological replicates from all conditions. The estimated correlation coefficients were visualized and further examined as described below (Additional file 2).

#### Construction of transcriptomic models

Transcriptomic models were constructed using Self-organizing map Harbouring Informative Nodes with Gene Ontology (SHIN+GO) [22, 43]. A self-organizing map (SOM) was trained with the normalized read count of all replicates described above. The matrix of 24 × 20 (480) was used with a rectangular shape (four neighbouring nodes). The epoch of 1000 times more than the map size was applied (i.e. 480,000, being 480 map size times 1000). The initial radius for SOM calculation was determined using a neighbour distance function in R kohonen package [44]. The following graphic outputs (Tatami maps) were produced and investigated: 1) genome-wide transcriptomic patterns of all biological replicates, and 2) genome-wide condition-specific transcriptomic patterns (Additional files 11 and 12). Similarly-regulated condition-specific genes were determined by fulfilling either of two criteria: 1) > 10.2 log2 reads (above 95th percentile of the entire transcribed genes used for transcriptomic models), or 2) > 2 log2 transcriptional differences of each growth point against growth point 1 (Additional file 11). Functional annotation sets were integrated into the constructed model using Carbohydrate Active Enzyme database (CAZy) [45], InterPro (IPR) [46]), the Gene Ontology (GO) [47], Kyoto Encyclopedia of Genes and Genomes (KEGG) [48], and EuKaryotic Orthologous Groups (KOG) [49] (Additional file 8). IPR, GO, KEGG, KOG, SignalP were obtained from Mycocosm, JGI (https://genome.jgi.doe.gov/Phaca1/Phaca1.home.html). CAZy annotations were obtained from AFMB, CNRS-Aix-Marseille University (http://www.cazy.org). All procedures were performed with the SHIN module of SHIN+GO.

### Carbohydrate content and composition

Samples were treated with 72% (*w*/w) H_2_SO_4_ (1 h, 30 °C) followed by hydrolysis with 1 M H_2_SO_4_ for 3 h at 100 °C. Hydrolysate was diluted 20 times and carbohydrate content and composition was determined by High Performance Anion Exchange chromatography (HPAEC) on a Dionex Ultimate ICS-3000 system (Thermo Scientific, Sunnyvale, CA, USA) equipped with an amperometric cell detector. Separation and quantification of monosaccharides was performed at a flow rate of 0.37 ml/min, with H_2_O as the eluent: The elution profile was as follows: 0–35 min 100% H_2_O; 35–42 min to 100% 0.2 M NaOH; 42–45 min to 100% H_2_O.

### Lignin content and composition measured using py-GC/MS (pyrolysis- gas chromatography/mass spectrometry)

Pyrolysis was performed with an EGA/PY-3030D Multi-shot pyrolyzer (Frontier Laboratories, New Ulm, MN, USA) equipped with an AS-1020E Autoshot auto-sampler as described previously by van Erven et al., (2017) [50]. The pyrolyzer was coupled to GC-MS using a Trace GC equipped with a DB-1701 fused-silica capillary column (30 m × 0.25 mm i.d. 0.25 μm film thickness) coupled to a DSQ-II mass spectrometer (both Thermo Scientific, Waltham, MA, USA). Pyrolysis, GC and MS settings were similar as previously described [51]. Samples were weighed using a XP6 excellence-plus microbalance (Mettler Toledo, Columbus, OH, USA). Pyrolysis of total biomass (70–80 μg) was performed at 500 °C for 1 min with an interface temperature of 300 °C. Pyrolysis products were injected on the column via split/splitless injection (at 250 °C) with a split ratio of 1:133 and helium was used as carrier gas with constant flow at 1.5 mL∙min^− 1^. The GC oven was programmed from 70 °C (2 min) to 270 °C at 5 °C∙min^− 1^ and held at 270 °C for 15 min. MS detection was used with EI at 70 eV, a source temperature of 250 °C, a scan range of *m/z* 50–550 and a scan rate of 4.0 scans/sec. Compounds were identified by comparing retention time and mass spectrum with standards, the NIST library and data published by Ralph and Hatfield [52].

For qualitative identification, pyrograms were processed by AMDIS software (version 2.71, NIST, USA). For identification and deconvolution the following software settings were used: minimum match factor at 60 with multiple identifications per compound, component width at 20, adjacent peak subtraction at two, resolution at high, sensitivity at very high and shape requirements at low. Compounds identified on the basis of reference standards were annotated by evaluation of retention time (± 0.1 min), reverse search (≥ 80) and simple search (≥ 30). Peak molar area was calculated as defined by Del Río et al., [53]. Lignin content was estimated on the basis of total area of lignin-derived pyrolysis products and compared to a wheat straw reference sample with known Klason lignin content (acid-insoluble lignin + acid-soluble lignin) [51]. All samples were analyzed in triplicate.

## Additional files

Additional file 1: Growth profile of *P. carnosa* on ground aspen and spruce. Cultivations were performed in Petri plates and were prepared in triplicate. Mycelia were harvested at five growth points (GP) for RNA extraction and sequencing. (TIFF 1783 kb)

Additional file 2: Correlation of transcriptomes among genes from 10 conditions with 2 replicates each. Left: Hierarchical clusters of biological replicates based on the distances of transcriptomic similarities. Right: Adjacent matrix of the correlation coefficients (*p* < 0.0001). AH/WH: Aspen/Spruce. 1_#/2_#/3_#/4_#/5_#: Growth points and followed by replicate IDs. (TIFF 550 kb)

Additional file 3: Molar carbohydrate composition (mol%) of Aspen (AH) and Spruce (WH) at growth point 1 and 5. Since different amounts of starting material were analyzed, similar relative quantities of carbohydrates between growth points indicates non-selective, simultaneous decay of biomass substrates. Rha, ramnosyl; Ara, arabinosyl; Xyl, xylosyl; Gal, galactosyl; Glc, glucosyl; * Man, mannosyl and glucuronosyl residues in traces. c- control sample; no fungal cultivation. (TIFF 3167 kb)

Additional file 4: Relative abundance of pyrolysis products and their structural features. AH: aspen heartwood, WH: white spruce heartwood, c: control sample; no fungal cultivation. Codes in brackets are used for peak annotation in Additional file 7. Since different amounts of starting material were analyzed, similar relative quantities of pyrolysis products between growth points indicates non-selective, simultaneous decay or transformation of biomass components. ^a^ miscellaneous ^b^ C_α_-oxygen ^c^ C_β_-oxygen ^d^ C_γ_-oxygen. (DOCX 26 kb)

Additional file 5: Lignin contents estimated by py-GC-MS for aspen and white spruce control (C) and *P. carnosa* grown at growth point 1 and 5. *significantly different from control at *P* ≤ 0.05. (TIFF 2495 kb)

Additional file 6: Counts per million (CPM) values for all sequences at all growth points and on each substrate (aspen heartwood – AH; white spruce heartwood – WH) is also shown. (XLSX 4817 kb)

Additional file 7: Transcript abundances (CPM values) of all annotated CAZymes and cytochrome P450s encoded by *P. carnosa*. (XLSX 586 kb)

Additional file 8: The annotations per protein IDs in 480 nodes. The nodes with high/differential transcriptions are labelled. The table also includes JGI protein IDs with following information. Log2 transformed normalized read counts of the genes averaged from the duplicates at all growth points; the log2 fold difference of the gene expression between time points with statistical significance (FDR adjusted *p* value < 0.05); functional annotation information on Carbohydrate Active Enzyme database (CAZy), InterPro (IPR), the Gene Ontology (GO), Kyoto Encyclopedia of Genes and Genomes (KEGG) and EuKaryotic Orthologous Groups (KOG), and SignalP for prediction of signal peptides. (XLSX 2752 kb)

Additional file 9: The distribution and density of normalized log2 transformed read counts of 11,796 genes from 10 conditions with 2 replicates each. AH/WH: Aspen/Spruce. 1_#/2_#/3_#/4_#/5_#: Growth points and followed by replicate IDs. (TIFF 1084 kb)

Additional file 10: The normalized log2 transformed read count of chitin synthase (11 genes) and NADH dehydrogenase (16 genes). AH/WH: Aspen/Spruce. 1_#/2_#/3_#/4_#/5_#: Growth points and followed by replicate IDs. (TIFF 1395 kb)

Additional file 11: Tatami maps showing the transcriptomic patterns of 20 replicates. AH/WH: Aspen/Spruce. 1_#/2_#/3_#/4_#/5_#: Growth points and followed by replicate IDs. The log2 read count of the replicates was overlaid onto the trained SOM. The vertical bar indicates the transcription levels. (TIFF 8620 kb)

Additional file 12: Condition-wise Tatami maps showing the averaged transcriptomic patterns from aspen/ spruce at five growth points. The averaged log2 read count of replicates grown in each condition was overlaid onto the trained SOM, representing the dynamics of genome-wide transcriptions corresponding to the conditions. (TIFF 6328 kb)

## Abbreviations

AA: Auxiliary activities
AOX: Alcohol oxidases
CAZy database: Carbohydrate Active Enzyme database
CAZyme: Carbohydrate-active enzyme
CPM: Count per million
CRO: GLOX/related copper radical oxidases
DREX: Distantly related to plant expansins
GH: Glycoside hydrolase
GLOX: Glyoxal oxidases
GO: Gene Ontology
GP: Growth points
HPAEC: High Performance Anion Exchange chromatography
KEGG: Kyoto Encyclopedia of Genes and Genomes
KOG: EuKaryotic Orthologous Groups
LiP: Lignin peroxidase
LOOL: Loosenin-like proteins
LPMO: Lytic polysaccharide monooxygenase
MnP: Manganese peroxidase
py-GC/MS: Pyrolysis- Gas chromatography/Mass spectrometry
SOM: Self-organizing map

## References

[1] Martinez D, Larrondo LF, Putnam N, Gelpke MDS, Huang K, Chapman J (2004). Genome sequence of the lignocellulose degrading fungus *Phanerochaete chrysosporium* strain RP78. Nat Biotechnol.

[2] Ohm RA, Riley R, Salamov A, Min B, Choi I-G, Grigoriev IV (2014). Genomics of wood-degrading fungi. Fungal Genet Biol.

[3] Burdsall HH Jr. A contribution to the taxonomy of the genus Phanerochaete (Corticiaceae, Aphyllophorales): J. Cramer; 1985.

[4] Suzuki H, MacDonald J, Syed K, Salamov A, Hori C, Aerts A (2012). Comparative genomics of the white-rot fungi, *Phanerochaete carnosa* and *P. chrysosporium*, to elucidate the genetic basis of the distinct wood types they colonize. BMC Genomics.

[5] Palonen H, Thomsen AB, Tenkanen M, Schmidt AS, Viikari L (2004). Evaluation of wet oxidation pretreatment for enzymatic hydrolysis of softwood. Appl Biochem Biotechnol.

[6] Zhu XJ Pan JY (2010). Woody biomass pretreatment for cellulosic ethanol production: technology and energy consumption evaluation. Bioresour Technol.

[7] Vanden Wymelenberg A, Gaskell J, Mozuch M, Splinter BonDurant S, Sabat G, Ralph J (2011). Significant alteration of gene expression in wood decay fungi *Postia placenta* and *Phanerochaete chrysosporium* by plant species. Appl Environ Microbiol.

[8] Korripally P, Hunt CG, Houtman CJ, Jones DC, Kitin PJ, Cullen D (2015). Regulation of gene expression during the onset of ligninolytic oxidation by *Phanerochaete chrysosporium* on spruce wood. Appl Environ Microbiol.

[9] Couturier M, Navarro D, Chevret D, Henrissat B, Piumi F, Ruiz-Dueñas FJ (2015). Enhanced degradation of softwood versus hardwood by the white-rot fungus *Pycnoporus coccineus*. Biotechnol Biofuels..

[10] Kuuskeri J, Häkkinen M, Laine P, Smolander O-P, Tamene F, Miettinen S (2016). Time-scale dynamics of proteome and transcriptome of the white-rot fungus *Phlebia radiata*: growth on spruce wood and decay effect on lignocellulose. Biotechnol Biofuels..

[11] Rytioja J, Hildén K, Hatakka A, Mäkelä MR (2014). Transcriptional analysis of selected cellulose-acting enzymes encoding genes of the white-rot fungus *Dichomitus squalens* on spruce wood and microcrystalline cellulose. Fungal Genet Biol.

[12] Rytioja J, Hildén K, Di Falco M, Zhou M, Aguilar-Pontes MV, Sietiö O (2017). The molecular response of the white-rot fungus *Dichomitus squalens* to wood and non-woody biomass as examined by transcriptome and exoproteome analyses. Environ Microbiol.

[13] Marinović M, Aguilar-Pontes MV, Zhou M, Miettinen O, de Vries RP, Mäkelä MR (2017). Temporal transcriptome analysis of the white-rot fungus *Obba rivulosa* shows expression of a constitutive set of plant cell wall degradation targeted genes during growth on solid spruce wood. Fungal Genet Biol.

[14] MacDonald J, Master ER (2012). Time-dependent profiles of transcripts encoding lignocellulose-modifying enzymes of the white rot fungus *Phanerochaete carnosa* grown on multiple wood substrates. Appl Environ Microbiol.

[15] MacDonald J, Doering M, Canam T, Gong Y, Guttman DS, Campbell MM (2011). Transcriptomic responses of the softwood-degrading white-rot fungus *Phanerochaete carnosa* during growth on coniferous and deciduous wood. Appl Environ Microbiol.

[16] Kersten P, Cullen D (2014). Copper radical oxidases and related extracellular oxidoreductases of wood-decay Agaricomycetes. Fungal Genet Biol.

[17] Vanden Wymelenberg A, Sabat G, Mozuch M, Kersten PJ, Cullen D, Blanchette RA (2006). Structure, organization, and transcriptional regulation of a family of copper radical oxidase genes in the lignin-degrading basidiomycete *Phanerochaete chrysosporium*. Appl Environ Microbiol.

[18] Couturier M, Ladevèze S, Sulzenbacher G, Ciano L, Fanuel M, Moreau C (2018). Lytic xylan oxidases from wood-decay fungi unlock biomass degradation. Nat Chem Biol Nature.

[19] Aspeborg H, Coutinho PM, Wang Y, Brumer H, Henrissat B (2012). Evolution, substrate specificity and subfamily classification of glycoside hydrolase family 5 (GH5). BMC Evol Biol.

[20] Behar H, Graham SW, Brumer H (2018). Comprehensive cross-genome survey and phylogeny of glycoside hydrolase family 16 members reveals the evolutionary origin of EG16 and XTH proteins in plant lineages. Plant J.

[21] Ichinose H (2013). Cytochrome P450 of wood-rotting basidiomycetes and biotechnological applications. Biotechnol Appl Biochem.

[22] Miyauchi S, Navarro D, Grigoriev IV, Lipzen A, Riley R, Chevret D (2016). Visual comparative omics of fungi for plant biomass deconstruction. Front Microbiol.

[23] Morel M, Meux E, Mathieu Y, Thuillier A, Chibani K, Harvengt L (2013). Xenomic networks variability and adaptation traits in wood decaying fungi. Microb Biotechnol.

[24] Mathieu Y, Prosper P, Favier F, Harvengt L, Didierjean C, Jacquot J-P (2013). Diversification of fungal specific class a glutathionetransferases in saprotrophic fungi. PLoS One.

[25] Quiroz-Castañeda RE, Martínez-Anaya C, Cuervo-Soto LI, Segovia L, Folch-Mallol JL (2011). Loosenin, a novel protein with cellulose-disrupting activity from *Bjerkandera adusta*. Microb Cell Factories.

[26] Cosgrove DJ (2000). Loosening of plant cell walls by expansins. Nature.

[27] Linder MB, Szilvay GR, Nakari-Setälä T, Penttilä ME (2005). Hydrophobins: the protein-amphiphiles of filamentous fungi. FEMS Microbiol Rev.

[28] Wösten HAB (2001). Hydrophobins: multipurpose proteins. Annu Rev Microbiol.

[29] Tanaka T, Nakayama M, Takahashi T, Nanatani K, Yamagata Y, Abe K (2017). Analysis of the ionic interaction between the hydrophobin RodA and two cutinases of *Aspergillus nidulans* obtained via an *Aspergillus oryzae* expression system. Appl Microbiol Biotechnol.

[30] Suzuki H, Vuong TV, Gong Y, Chan K, Ho CY, Master ER (2014). Sequence diversity and gene expression analyses of expansin-related proteins in the white-rot basidiomycete, *Phanerochaete carnosa*. Fungal Genet Biol.

[31] Aimanianda V, Simenel C, Garnaud C, Clavaud C, Tada R, Barbin L (2017). The dual activity responsible for the elongation and branching of β-(1,3)-glucan in the fungal cell wall. MBio.

[32] Blanchette RA, Krueger EW, Haight JE, Aktar M, Akin DE (1997). Cell wall alterations in loblolly pine wood decayed by the white-rot fungus, *Ceriporiopsis subvermispora*. J Biotechnol.

[33] Tanaka H, Itakura S, Enoki A (1999). Hydroxyl radical generation by an extracellular low-molecular weight substance and phenol oxidase activity during wood degradation by the white-rot basidiomycete *Phanerochaete chrysosporium*. J Biotechnol.

[34] Tanaka H, Yoshida G, Baba Y, Matsumura K, Wasada H, Murata J (2007). Characterization of a hydroxyl-radical producing glycoprotein and its presumptive genes from the white-rot basidiomycete *Phanerochaete chrysosporium*. J Biotechnol.

[35] Wu B, Gaskell J, Held BW, Toapanta C, Vuong T, Ahrendt S, et al. Substrate-specific differential gene expression and RNA editing in the brown rot fungus *Fomitopsis pinicola*. Appl Environ Microbiol. 2018;84(16). 10.1128/AEM.00991-18 PMC6070754 29884757

[36] Mouyna I, Aimanianda V, Hartl L, Prevost MC, Sismeiro O, Dillies MA (2016). GH16 and GH81 family β-(1,3)-glucanases in *Aspergillus fumigatus* are essential for conidial cell wall morphogenesis. Cell Microbiol.

[37] Roemer T, Bussey H (1991). Yeast beta-glucan synthesis: KRE6 encodes a predicted type II membrane protein required for glucan synthesis in vivo and for glucan synthase activity in vitro. Proc Natl Acad Sci U S A.

[38] You S, Tu T, Zhang L, Wang Y, Huang H, Ma R (2016). Improvement of the thermostability and catalytic efficiency of a highly active β-glucanase from *Talaromyces leycettanus* JCM12802 by optimizing residual charge-charge interactions. Biotechnol Biofuels..

[39] Hori C, Gaskell J, Igarashi K, Kersten P, Mozuch M, Samejima M (2014). Temporal alterations in the secretome of the selective ligninolytic fungus *Ceriporiopsis subvermispora* during growth on aspen wood reveal this organism’s strategy for degrading lignocellulose. Appl Environ Microbiol.

[40] Peng M, Aguilar-Pontes MV, Hainaut M, Henrissat B, Hildén K, Mäkelä MR (2018). Comparative analysis of basidiomycete transcriptomes reveals a core set of expressed genes encoding plant biomass degrading enzymes. Fungal Genet Biol.

[41] Robinson MD, McCarthy DJ, Smyth GK (2010). edgeR: a Bioconductor package for differential expression analysis of digital gene expression data. Bioinformatics.

[42] Love MI, Huber W, Anders S (2014). Moderated estimation of fold change and dispersion for RNA-seq data with DESeq2. Genome Biol.

[43] Miyauchi S, Navarro D, Grisel S, Chevret D, Berrin J-G, Rosso M-N (2017). The integrative omics of white-rot fungus *Pycnoporus coccineus* reveals co-regulated CAZymes for orchestrated lignocellulose breakdown. PLoS One.

[44] Wehrens R, Buydens LMC (2007). Self- and super-organizing maps in R: the kohonen package. J Stat Softw.

[45] Levasseur A, Drula E, Lombard V, Coutinho PM, Henrissat B (2013). Expansion of the enzymatic repertoire of the CAZy database to integrate auxiliary redox enzymes. Biotechnol Biofuels.

[46] Finn RD, Attwood TK, Babbitt PC, Bateman A, Bork P, Bridge AJ (2017). InterPro in 2017—beyond protein family and domain annotations. Nucleic Acids Res.

[47] Consortium TGO (2015). Gene ontology consortium: going forward. Nucleic Acids Res.

[48] Ogata H, Goto S, Sato K, Fujibuchi W, Bono H, Kanehisa M (1999). KEGG: Kyoto encyclopedia of genes and genomes. Nucleic Acids Res.

[49] Tatusov RL, Fedorova ND, Jackson JD, Jacobs AR, Kiryutin B, Koonin EV (2003). The COG database: an updated version includes eukaryotes. BMC Bioinformatics.

[50] van Erven G, de Visser R, Merkx DWH, Strolenberg W, de Gijsel P, Gruppen H (2017). Quantification of lignin and its structural features in plant biomass using 13C lignin as internal standard for pyrolysis-GC-SIM-MS. Anal Chem.

[51] Jurak E, Punt AM, Arts W, Kabel MA, Gruppen H (2015). Fate of carbohydrates and lignin during composting and mycelium growth of *Agaricus bisporus* on wheat straw based compost. PLoS One.

[52] Ralph J, Hatfield RD (1991). Pyrolysis GC-MS characterization of forage materials. J Agric Food Chem.

[53] del Río JC, Rencoret J, Prinsen P, Martínez ÁT, Ralph J, Gutiérrez A (2012). Structural characterization of wheat straw lignin as revealed by analytical pyrolysis, 2D-NMR, and reductive cleavage methods. J Agric Food Chem.

