# Supplementary figures and images for: Dynamics of the Phanerochaete carnosa transcriptome during growth on aspen and spruce
Source: BMC Genomics. 2018 Nov 13;19:815. doi: 10.1186/s12864-018-5210-z (PMC6234650; doi:10.1186/s12864-018-5210-z)

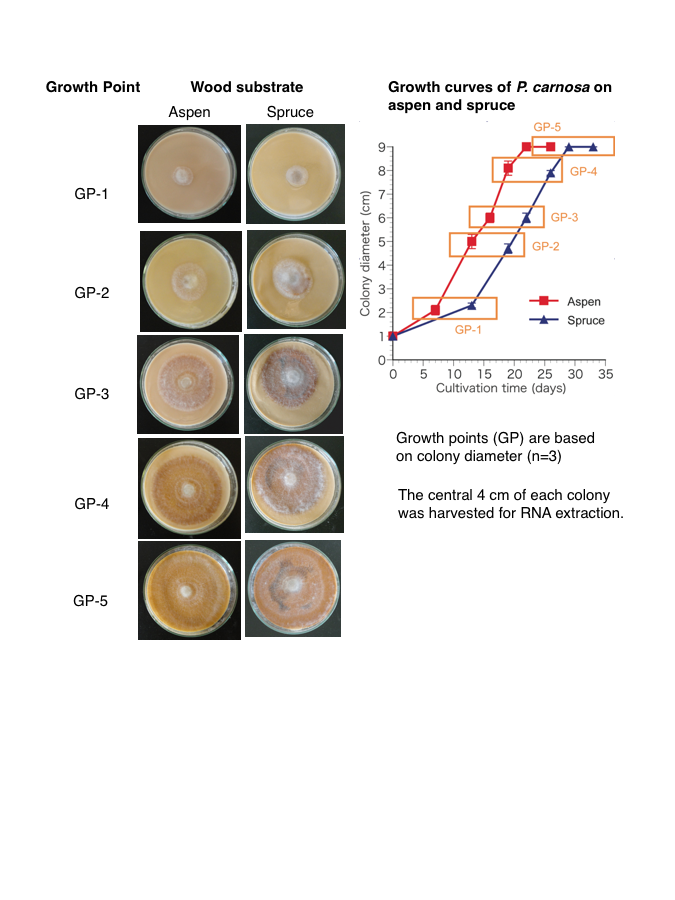

Supplement: Supplementary file 1 — Growth profile of P. carnosa on ground aspen and spruce. Cultivations were performed in Petri plates and were prepared in triplicate. Mycelia were harvested at five growth points (GP) for RNA extraction and sequencing. (TIFF 1783 kb) [file 12864_2018_5210_MOESM1_ESM.tiff]

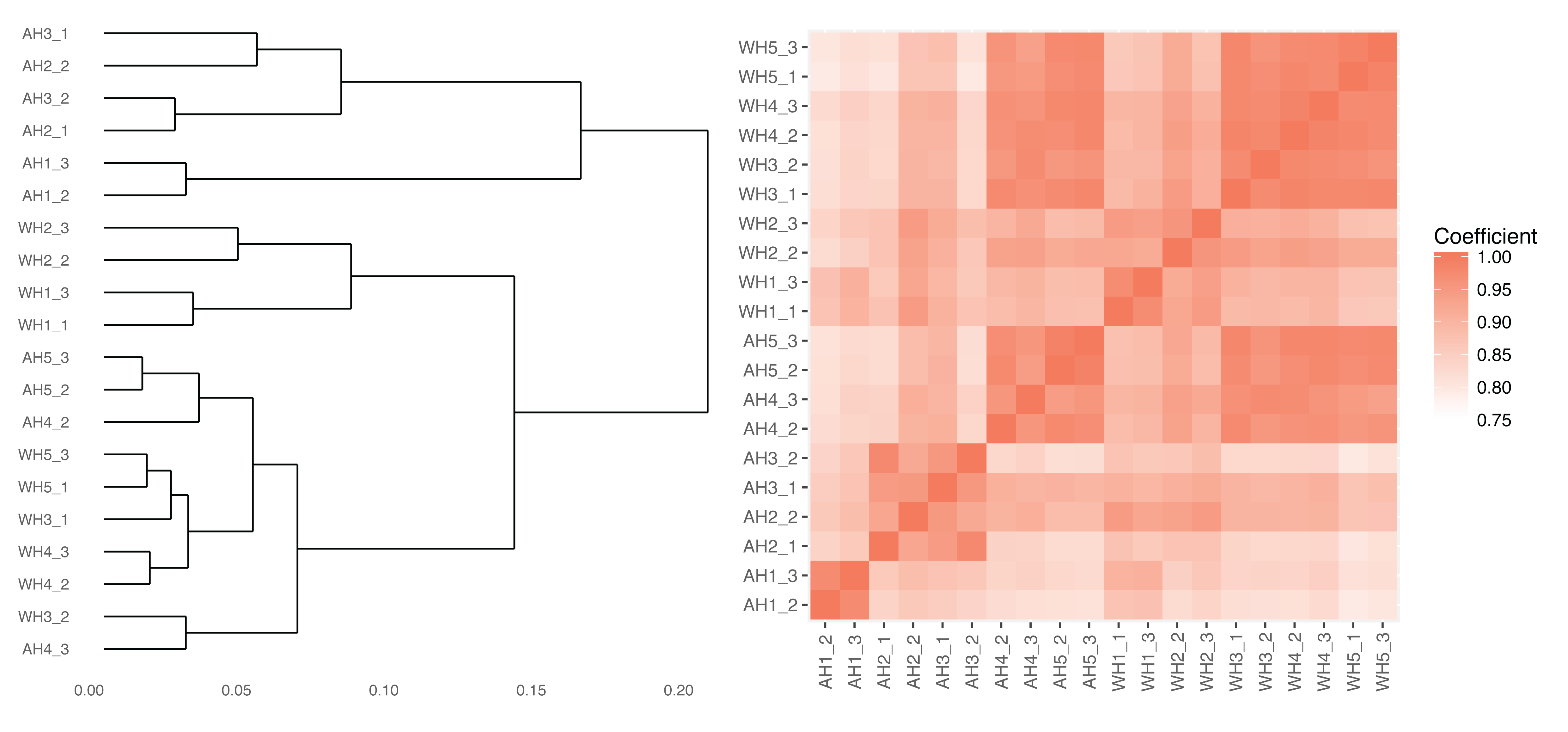

Supplement: Supplementary file 2 — Correlation of transcriptomes among genes from 10 conditions with 2 replicates each. Left: Hierarchical clusters of biological replicates based on the distances of transcriptomic similarities. Right: Adjacent matrix of the correlation coefficients (p < 0.0001). AH/WH: Aspen/Spruce. 1_#/2_#/3_#/4_#/5_#: Growth points and followed by replicate IDs. (TIFF 550 kb) [file 12864_2018_5210_MOESM2_ESM.tiff]

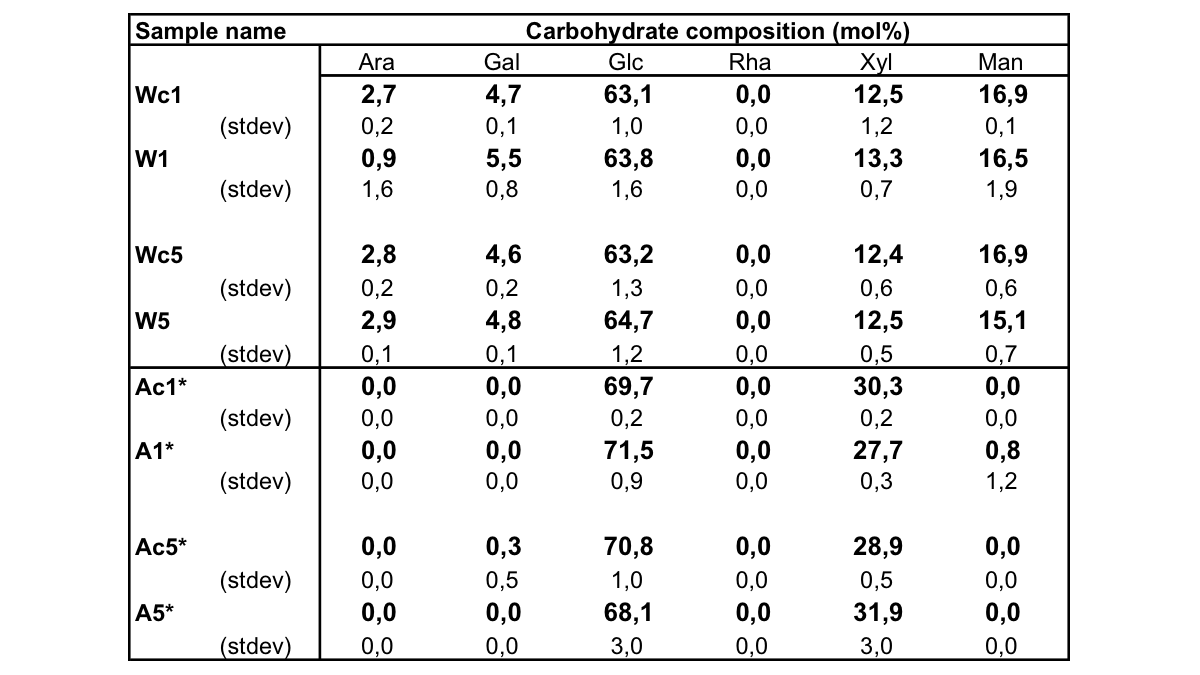

Supplement: Supplementary file 3 — Molar carbohydrate composition (mol%) of Aspen (AH) and Spruce (WH) at growth point 1 and 5. Since different amounts of starting material were analyzed, similar relative quantities of carbohydrates between growth points indicates non-selective, simultaneous decay of biomass substrates. Rha, ramnosyl; Ara, arabinosyl; Xyl, xylosyl; Gal, galactosyl; Glc, glucosyl; * Man, mannosyl and glucuronosyl residues in traces. c- control sample; no fungal cultivation. (TIFF 3167 kb) [file 12864_2018_5210_MOESM3_ESM.tiff]

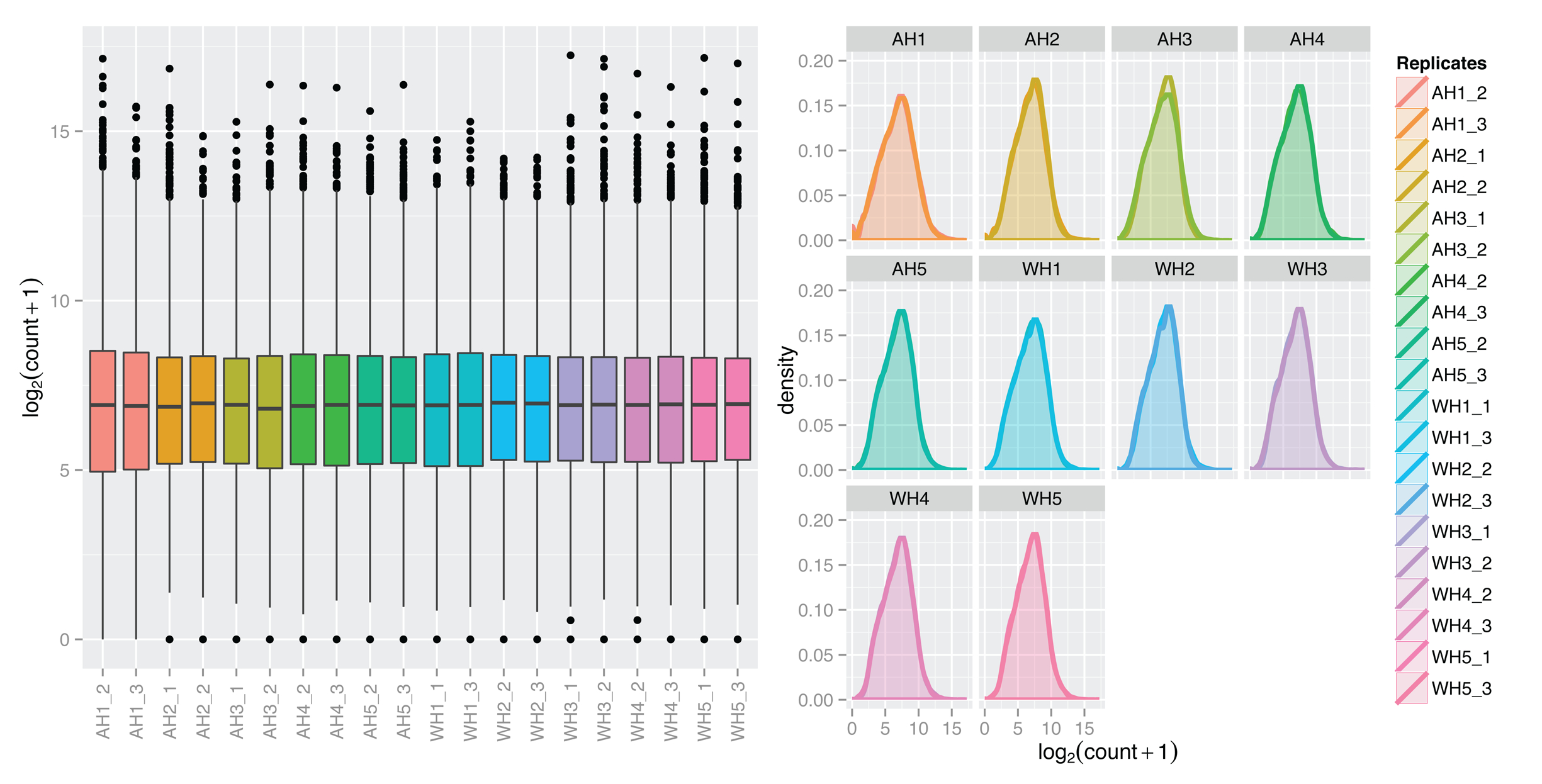

Supplement: Supplementary file 9 — The distribution and density of normalized log2 transformed read counts of 11,796 genes from 10 conditions with 2 replicates each. AH/WH: Aspen/Spruce. 1_#/2_#/3_#/4_#/5_#: Growth points and followed by replicate IDs. (TIFF 1084 kb) [file 12864_2018_5210_MOESM9_ESM.tiff]

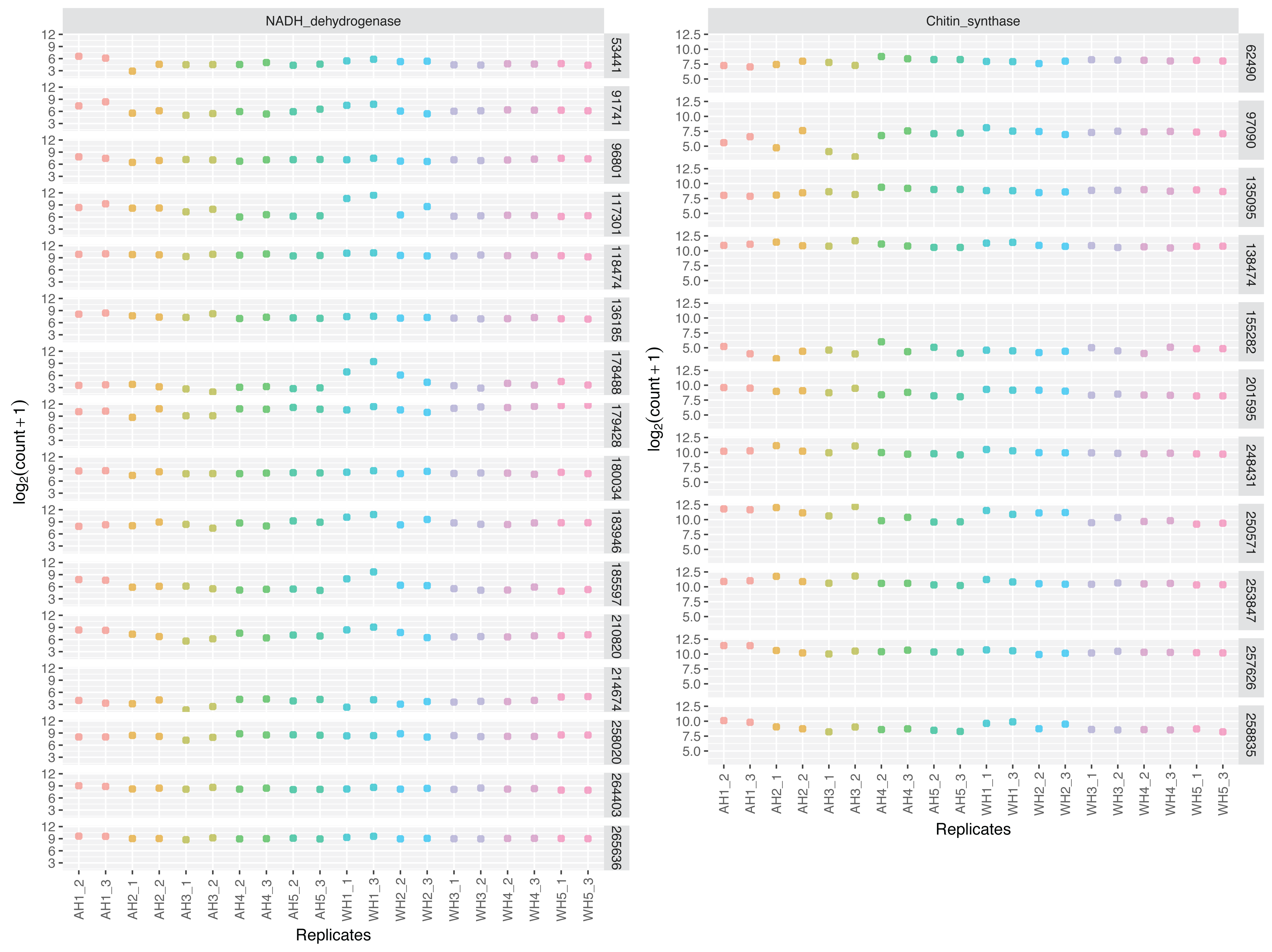

Supplement: Supplementary file 10 — The normalized log2 transformed read count of chitin synthase (11 genes) and NADH dehydrogenase (16 genes). AH/WH: Aspen/Spruce. 1_#/2_#/3_#/4_#/5_#: Growth points and followed by replicate IDs. (TIFF 1395 kb) [file 12864_2018_5210_MOESM10_ESM.tiff]

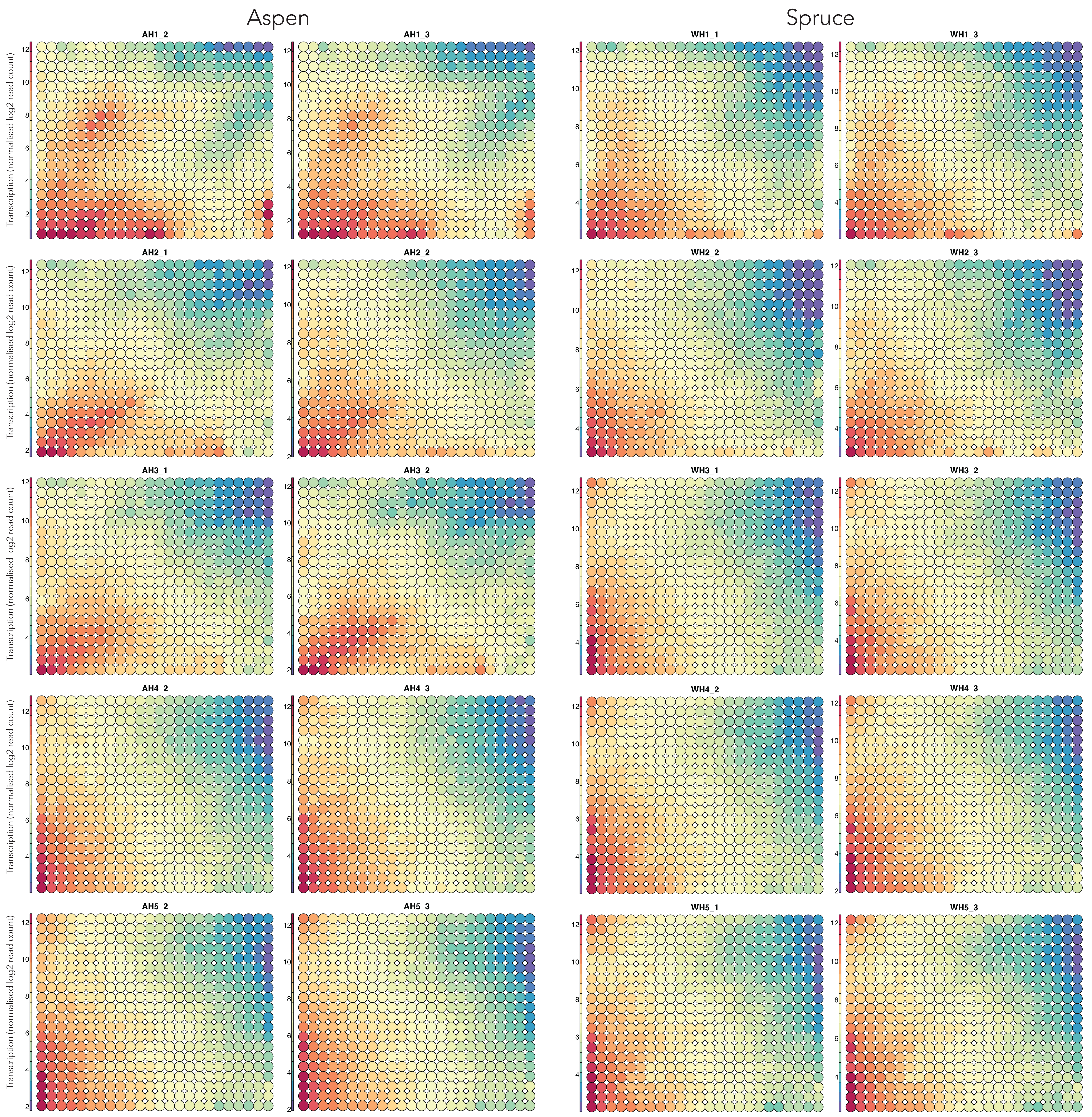

Supplement: Supplementary file 11 — Tatami maps showing the transcriptomic patterns of 20 replicates. AH/WH: Aspen/Spruce. 1_#/2_#/3_#/4_#/5_#: Growth points and followed by replicate IDs. The log2 read count of the replicates was overlaid onto the trained SOM. The vertical bar indicates the transcription levels. (TIFF 8620 kb) [file 12864_2018_5210_MOESM11_ESM.tiff]

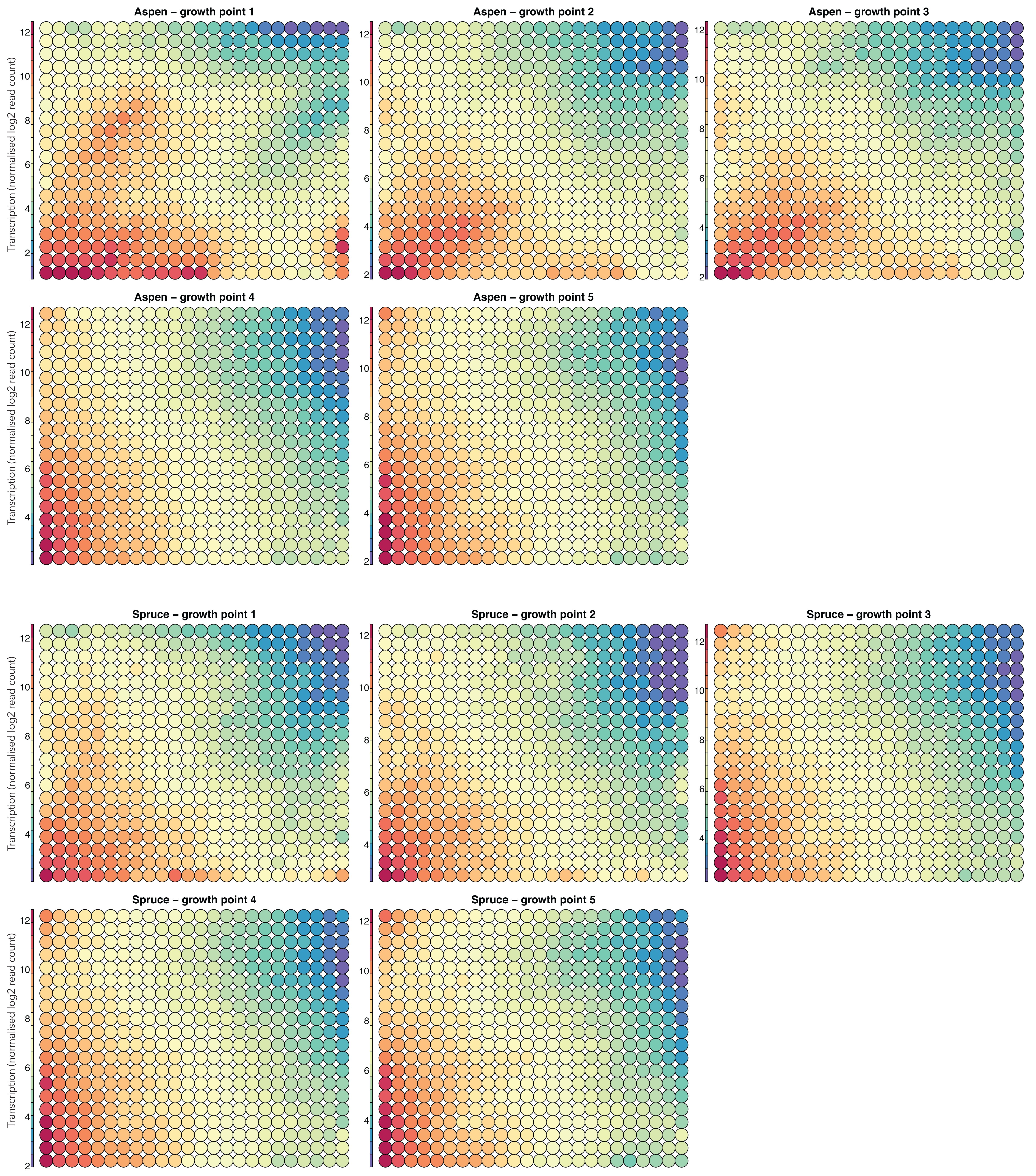

Supplement: Supplementary file 12 — Condition-wise Tatami maps showing the averaged transcriptomic patterns from aspen/ spruce at five growth points. The averaged log2 read count of replicates grown in each condition was overlaid onto the trained SOM, representing the dynamics of genome-wide transcriptions corresponding to the conditions. (TIFF 6328 kb) [file 12864_2018_5210_MOESM12_ESM.tiff]
