# Supplementary material for: Dynamics of the Phanerochaete carnosa transcriptome during growth on aspen and spruce
Source: BMC Genomics. 2018 Nov 13;19:815. doi: 10.1186/s12864-018-5210-z (PMC6234650; doi:10.1186/s12864-018-5210-z)
Supplement: Supplementary file 4 — Relative abundance of pyrolysis products and their structural features. AH: aspen heartwood, WH: white spruce heartwood, c: control sample; no fungal cultivation. Codes in brackets are used for peak annotation in Additional file 7. Since different amounts of starting material were analyzed, similar relative quantities of pyrolysis products between growth points indicates non-selective, simultaneous decay or transformation of biomass components. a miscellaneous b Cα-oxygen c Cβ-oxygen d Cγ-oxygen. (DOCX 26 kb) [file 12864_2018_5210_MOESM4_ESM.docx]

**Additional file 4.** Relative abundance of pyrolysis products and their structural features. AH:

aspen heartwood, WH: white spruce heartwood, c: control sample; no fungal cultivation.

^a^ miscellaneous; ^b^ Cα-oxygen; ^c^ Cβ-oxygen; ^d^ Cγ-oxygen

| **#** | **Compound** | **CAS** | **Retention time (min)** | **Mw (g∙mol^-1^)** | **Structural**  **feature** | **AHc1** | **AH1** | **AHc5** | **AH5** | **WHc1** | **WH1** | **WHc5** | **WH5** |
| --- | --- | --- | --- | --- | --- | --- | --- | --- | --- | --- | --- | --- | --- |
| 1 | phenol | 108952 | 9.89 | 94 | H, unsub. | 11.1 | 9.4 | 9.7 | 8.0 | 1.9 | 1.9 | 1.8 | 1.9 |
| 2 | 2-methylphenol | 95487 | 11.13 | 108 | H, methyl | 0.4 | 0.4 | 0.4 | 0.4 | 0.5 | 0.5 | 0.5 | 0.5 |
| 3 | 4-methylphenol | 106445 | 12.07 | 108 | H, methyl | 0.3 | 0.2 | 0.1 | 0.3 | 0.7 | 0.9 | 0.8 | 0.9 |
| 4 | 4-ethylphenol | 123079 | 14.33 | 122 | H, ethyl | 0.0 | 0.0 | 0.0 | 0.1 | 0.1 | 0.0 | 0.1 | 0.1 |
| 5 | 4-vinylphenol | 2628173 | 16.57 | 120 | G, methyl | 0.2 | 0.2 | 0.2 | 0.1 | 0.6 | 0.6 | 0.5 | 0.6 |
| 6 | hydroquinone | 123319 | 20.62 | 110 | H, misc.^a^ | 1.9 | 1.9 | 1.9 | 2.2 | 1.7 | 1.8 | 1.7 | 1.2 |
| 7 | 4-hydroxybenzaldehyde | 123080 | 22.81 | 122 | H, C_α_-O^b^ | 0.0 | 0.0 | 0.0 | 0.0 | 0.2 | 0.1 | 0.2 | 0.2 |
| 8 | guaiacol | 90051 | 10.12 | 124 | G, unsub. | 8.0 | 7.8 | 7.4 | 7.3 | 18.1 | 18.2 | 18.2 | 17.8 |
| 9 | 4-methylguaiacol | 93516 | 12.79 | 138 | G, methyl | 1.4 | 1.4 | 1.3 | 1.5 | 7.1 | 7.9 | 7.2 | 7.6 |
| 10 | 4-ethylguaiacol | 2785899 | 14.94 | 152 | G, ethyl | 0.5 | 0.5 | 0.4 | 0.4 | 1.2 | 1.3 | 1.2 | 1.2 |
| 11 | 4-vinylguaiacol | 7786610 | 16.40 | 150 | G, vinyl | 4.6 | 4.4 | 4.5 | 4.4 | 12.4 | 12.7 | 12.4 | 12.9 |
| 12 | eugenol | 97530 | 17.00 | 164 | G, misc. | 0.3 | 0.3 | 0.3 | 0.3 | 0.9 | 0.9 | 0.9 | 0.9 |
| 13 | 4-propylguaiacol | 2785877 | 17.10 | 166 | G, misc. | 0.2 | 0.2 | 0.1 | 0.2 | 0.6 | 0.6 | 0.5 | 0.6 |
| 14 | *cis*-isoeugenol | 97541 | 18.35 | 164 | G, misc. | 0.2 | 0.2 | 0.2 | 0.2 | 0.5 | 0.5 | 0.5 | 0.5 |
| 15 | *trans*-isoeugenol | 97541 | 19.62 | 164 | G, misc. | 1.2 | 1.2 | 1.2 | 1.3 | 4.3 | 4.6 | 4.4 | 4.7 |
| 16 | vanillin | 121335 | 20.09 | 152 | G, C_α_-O | 1.5 | 1.6 | 1.5 | 1.7 | 4.3 | 4.4 | 4.1 | 4.7 |
| 17 | 4-propyneguaiacol | - | 20.33 | 162 | G, misc. | 0.2 | 0.2 | 0.2 | 0.2 | 0.3 | 0.3 | 0.3 | 0.3 |
| 18 | 4-alleneguaiacol | - | 20.59 | 162 | G, misc. | 0.3 | 0.1 | 0.2 | 0.0 | 0.2 | 0.3 | 0.2 | 0.2 |
| 19 | homovanillin | 5703242 | 21.53 | 166 | G, C_β_-O^c^ | 1.0 | 1.0 | 1.0 | 0.9 | 2.1 | 2.1 | 2.0 | 1.8 |
| 20 | acetovanillone | 498022 | 22.00 | 166 | G, C_α_-O | 1.0 | 1.0 | 1.0 | 1.1 | 2.7 | 2.7 | 2.6 | 2.9 |
| 21 | guaiacylacetone | 2503460 | 23.21 | 180 | G, C_β_-O | 1.3 | 1.3 | 1.2 | 1.3 | 2.7 | 2.5 | 2.7 | 2.8 |
| 22 | homovanillyl alcohol | 2380781 | 23.31 | 168 | G, C_β_-O | 0.0 | 0.0 | 0.0 | 0.0 | 0.1 | 0.1 | 0.1 | 0.1 |
| 23 | propiovanillone | 1835149 | 23.80 | 180 | G, misc. | 0.1 | 0.1 | 0.1 | 0.1 | 0.3 | 0.4 | 0.3 | 0.4 |
| 24 | guaiacyl vinyl ketone | - | 24.19 | 178 | G, C_α_-O | 0.2 | 0.1 | 0.2 | 0.3 | 0.6 | 0.6 | 0.5 | 0.6 |
| 25 | dihydroconiferyl alcohol | 2305137 | 25.88 | 182 | G, misc. | 0.0 | 0.0 | 0.0 | 0.0 | 1.7 | 1.7 | 1.7 | 1.8 |
| 26 | *cis*-coniferyl-alcohol | 458355 | 26.54 | 180 | G, C_γ_-O^d^ | 0.5 | 0.6 | 0.6 | 0.6 | 1.3 | 1.2 | 1.3 | 1.3 |
| 27 | *trans*-coniferyl-alcohol | 458355 | 28.25 | 180 | G, C_γ_-O | 12.8 | 13.0 | 13.7 | 14.1 | 29.8 | 28.1 | 30.1 | 28.3 |
| 28 | *trans*-coniferaldehyde | 458366 | 28.62 | 178 | G, C_γ_-O | 0.7 | 0.7 | 0.8 | 0.8 | 3.3 | 3.2 | 3.1 | 3.3 |
| 29 | syringol | 91101 | 17.75 | 154 | S, unsub. | 13.3 | 14.0 | 12.9 | 12.9 | 0.0 | 0.0 | 0.0 | 0.0 |
| 30 | 4-methylsyringol | 6638057 | 19.98 | 168 | S, methyl | 2.2 | 2.4 | 2.3 | 2.7 | 0.0 | 0.0 | 0.0 | 0.0 |
| 31 | 4-ethylsyringol | 14059928 | 21.71 | 182 | S, ethyl | 0.6 | 0.6 | 0.5 | 0.5 | 0.0 | 0.0 | 0.0 | 0.0 |
| 32 | 4-vinylsyringol | 28343228 | 23.03 | 180 | S, vinyl | 11.0 | 10.8 | 11.1 | 10.4 | 0.0 | 0.0 | 0.0 | 0.0 |
| 33 | 4-allylsyringol | 6627889 | 23.44 | 194 | S, misc. | 0.6 | 0.7 | 0.6 | 0.7 | 0.0 | 0.0 | 0.0 | 0.0 |
| 34 | *cis*-4-propenylsyringol | 26624135 | 24.56 | 194 | S, misc. | 0.3 | 0.3 | 0.3 | 0.3 | 0.0 | 0.0 | 0.0 | 0.0 |
| 35 | 4-propynesyringol | - | 25.21 | 192 | S, misc. | 0.2 | 0.2 | 0.2 | 0.2 | 0.0 | 0.0 | 0.0 | 0.0 |
| 36 | 4-allenesyringol | - | 25.40 | 192 | S, misc. | 0.2 | 0.2 | 0.2 | 0.2 | 0.0 | 0.0 | 0.0 | 0.0 |
| 37 | *trans*-4-propenylsyringol | 26624135 | 25.86 | 194 | S, misc. | 2.7 | 3.0 | 2.8 | 3.2 | 0.0 | 0.0 | 0.0 | 0.0 |
| 38 | syringaldehyde | 134963 | 26.46 | 182 | S, C_α_-O | 2.7 | 2.9 | 2.9 | 3.0 | 0.0 | 0.0 | 0.0 | 0.0 |
| 39 | acetosyringone | 2478388 | 27.90 | 196 | S, C_α_-O | 1.5 | 1.8 | 1.6 | 1.8 | 0.0 | 0.0 | 0.0 | 0.0 |
| 40 | syringylacetone | 19037582 | 28.82 | 210 | S, C_β_-O | 2.4 | 2.5 | 2.5 | 2.5 | 0.0 | 0.0 | 0.0 | 0.0 |
| 41 | syringoyl acetaldehyde | - | 29.61 | 224 | S, C_α_-O, C_γ_-O | 0.1 | 0.1 | 0.1 | 0.2 | 0.0 | 0.0 | 0.0 | 0.0 |
| 42 | syringyl vinyl ketone | - | 29.68 | 208 | S, C_α_-O | 0.1 | 0.1 | 0.1 | 0.2 | 0.0 | 0.0 | 0.0 | 0.0 |
| 43 | *cis*-sinapyl-alcohol | 537337 | 31.76 | 210 | S, C_γ_-O | 0.5 | 0.6 | 0.6 | 0.6 | 0.0 | 0.0 | 0.0 | 0.0 |
| 44 | *trans*-sinapyl-alcohol | 537337 | 33.47 | 210 | S, C_γ_-O | 10.9 | 10.5 | 11.8 | 11.5 | 0.0 | 0.0 | 0.0 | 0.0 |
| 45 | *trans*-sinapaldehyde | 4206580 | 33.68 | 208 | S, C_γ_-O | 1.1 | 1.1 | 1.2 | 1.3 | 0.0 | 0.0 | 0.0 | 0.0 |
